# Human 45,X Fibroblast Transcriptome Reveals Distinct Differentially Expressed Genes Including Long Noncoding RNAs Potentially Associated with the Pathophysiology of Turner Syndrome

**DOI:** 10.1371/journal.pone.0100076

**Published:** 2014-06-16

**Authors:** Shriram N. Rajpathak, Shamsudheen Karuthedath Vellarikkal, Ashok Patowary, Vinod Scaria, Sridhar Sivasubbu, Deepti D. Deobagkar

**Affiliations:** 1 Centre of Advanced Studies, Department of Zoology, University of Pune, Pune, India; 2 Genomics and Molecular medicine, CSIR Institute of Genomics and Integrative Biology, Delhi, India; 3 GN Ramachandran Knowledge Centre for Genome Informatics, CSIR Institute of Genomics and Integrative Biology, Delhi, India; 4 Academy of Scientific and Innovative Research (AcSIR), Anusandhan Bhawan, 2 Rafi Marg, New Delhi, India; Florida State University, United States of America

## Abstract

Turner syndrome is a chromosomal abnormality characterized by the absence of whole or part of the X chromosome in females. This X aneuploidy condition is associated with a diverse set of clinical phenotypes such as gonadal dysfunction, short stature, osteoporosis and Type II diabetes mellitus, among others. These phenotypes differ in their severity and penetrance among the affected individuals. Haploinsufficiency for a few X linked genes has been associated with some of these disease phenotypes. RNA sequencing can provide valuable insights to understand molecular mechanism of disease process. In the current study, we have analysed the transcriptome profiles of human untransformed 45,X and 46,XX fibroblast cells and identified differential expression of genes in these two karyotypes. Functional analysis revealed that these differentially expressing genes are associated with bone differentiation, glucose metabolism and gonadal development pathways. We also report differential expression of lincRNAs in X monosomic cells. Our observations provide a basis for evaluation of cellular and molecular mechanism(s) in the establishment of Turner syndrome phenotypes.

## Introduction

In eutherian mammals and marsupials, differential gene expression in a sex chromosome is achieved by a phenomenon called dosage compensation, which is mediated through inactivation of one of the two X chromosomes in females [1]. Maintenance of the dosage balance is an important aspect for the optimal biological function of an organism. A number of reports have suggested that alteration of gene expression in dosage sensitive genes could potentially lead to biological implications and in some cases disease phenotypes [2], [3]. The gene dosage imbalance could be contributed by copy number changes including duplications/deletions and copy-neutral changes including imprinting and inactivation [4], [5]. The spectrum of these changes could also be highly variable ranging from a few kilo bases in the genome to parts or whole of the chromosomes in some cases. Most of the large chromosomal abnormalities are thought to lead to first trimester abortions or foetal death, without manifesting a live offspring. A number of disease conditions including cancer [6]–[8], genetic disorders such as Williams syndrome [9] and Dyskeratosis congenita [10] have also been correlated with gene dosage imbalances. In humans, aneuploidy conditions have been extensively studied with respect to common chromosomal abnormality syndromes represented by either the presence of an extra chromosome (Down’s syndrome, Klinefelter’s syndrome) or absence of a chromosome (Turner syndrome).

Turner syndrome (TS) is a complex genetic disorder caused by a complete or partial monosomy of the X chromosome. Turner syndrome occurs in approximately 1 in every 1,800–2,500 live female births [11]. It is estimated that almost 99% of fetuses affected with Turner syndrome end up in abortions/fetal death [12]. Classical Turner syndrome patients exhibit characteristic features like short stature, ovarian dysfunction, osteoporosis, and diabetes mellitus type II, apart from neurological features [13], [14]. The disease is characterized by a high variability in clinical presentation and penetrance of the phenotypes. It has been well established that haploinsufficiency of the X linked genes [15]–[17] that escape X inactivation [18] is one of the major factors responsible for these clinical phenotypes. Global gene expression profiling techniques like microarrays have been previously used to study differential gene expression patterns in human aneuploidy conditions. Nevertheless, these studies have been largely limited to Down syndrome and tissues as varied as fibroblasts [19], whole blood [20] and brain [21] have been studied. A few studies have reported altered expression profiles for X linked [22] and autosomal genes [23] in Klinefelter syndrome.

In a recent study, Zhang *et. al* [24] have used RNA-sequencing of human induced pluripotent stem cells (iPSCs), to show that aneuploidic conditions have specific effects on the transcriptome. Such conditions induce alterations in expression levels of certain genes that may give rise to multiple pathological features of a disease. The authors suggested that differentially expressed genes in Turner syndrome are related to neuronal development and cancer related pathways. Earlier studies have also suggested that the serum levels of insulin-like growth factor (IGF), insulin-like growth factor binding protein-3 (IGFBP3), anti-Müllerian hormone (AMH) and androgens are altered in individuals with Turner syndrome as compared to normal females [25]–[27]. Bakalov *et. al* [28] reported that 45,X individuals have a significantly altered expression of many X linked as well as autosomal genes compared to 46,X,i(X)q individuals. The authors also suggested that change in expression of autosomal genes like insulin receptor substrate 2 (IRS2) and IGF in Turner syndrome could have an effect in modulating their increased susceptibility to type 2 Diabetes mellitus. In addition to genome wide alterations in gene expression, there are reports, that also indicate alterations in epigenetic signatures in Down syndrome [29] and in Turner syndrome [30]. Our earlier analysis has suggested a possible link between mis-regulation of DNA methylation in Turner syndrome [30], [31].

In the present study, we have analysed the transcriptome of un-transformed fibroblasts with distinct chromosomal aneuploidy (45,X), characteristic of Turner syndrome and a normal female (46,XX). Our analysis reports a subset of genes including long non-coding RNAs such as X inactive specific transcript (XIST) that are differentially expressed and associated with the 45,X aneuploidy. These differentially expressed genes are associated with various biological processes deregulated in Turner syndrome and potentially provide an explanation for a subset of the phenotypic complexity associated with Turner syndrome.

## Results

### Transcriptome Data Generation and Analysis

Poly-A RNA was obtained from the 45,X and 46,XX cells. RNA sequence reads were generated using paired end sequencing method. A total of approximately 21 million (45,X) and over 29 million (46,XX) raw paired end sequence reads were generated using Illumina Genome Analyser IIx. After quality filtering over 97.5% and 97.4% of the reads from the 45,X and the 46,XX fibroblasts respectively were used for analysis. The sequence reads were further mapped back to the human reference genome (hg19 version of the Reference Human Genome). RNA sequencing, mapping and analysis was done as per the protocol described in the materials and methods section. Over 97% of the reads could be mapped to the human reference genome for both the datasets. The sequencing results and data analysis is summarised in Table 1. The mapped reads were further processed using cufflinks to annotate expression values based on the GENCODE transcripts and gene loci annotations. Briefly, the reads mapping from each of the dataset for the corresponding gene annotations were calculated as Fragments Per Kilo base of transcript per Million mapped reads (FPKM).

**Table 1:** Summary of RNA-seq data generated and mapping statistics.

DatasetFiltering	Raw readsgenerated (Paired)	Reads afterQC filtering(Paired)	Unmappedreads	MappedPercentage
45,X	2,11,25,165	1,60,28,094	7,88,657	97.54%
46,XX	2,90,67,334	2,11,18,131	10,98,054	97.40%

Total number of reads generated and mapped after quality filtering for both 45,X and 46,XX human fibroblast cells.

### Differential Expression of Genes

**Figure 1:**
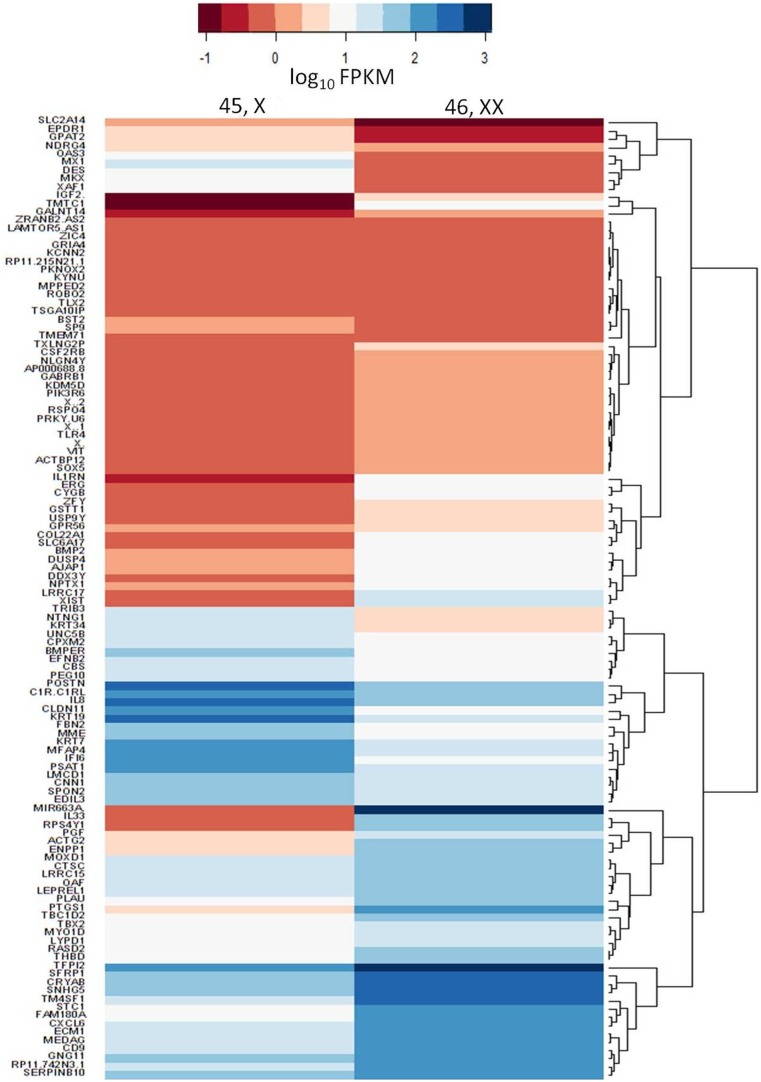
Heat Map depicting the expression levels 116 significantly differentially expressed genes in 45,X and 46,XX fibroblast cells.

The expression values were further normalised using quantile normalisation method as implemented in cuffdiff [32]. The genes that show significant differences in expression were retrieved for further analysis. A total of 116 genes were found to be differentially expressed between the two sets (expression profiles are summarised in Figure 1 and Table S1). Of the 116 differentially expressed genes identified from our analysis, 8 genes were annotated to the Y chromosome. Out of these 8 genes, 4 are located on the pseudo-autosomal region. It is known that pseudo-autosomal genes have homologues on both X and Y chromosomes and they escape X inactivation. The remaining 4 genes have significant (90–91%) similarity to autosomal genes, and therefore were removed from further analysis. Out of the remaining 108 genes, 77 genes that showed at least 2 fold expression change were taken up for further functional annotation. Among these 77 genes, 23% (>5 fold) and 18% (>2 fold) were significantly up regulated in 45,X cells while 35% (>5 fold), 24% (>2 fold) were significantly up regulated in 46,XX cells. A similar finding was observed by Zhang *et. al* [24] where X monosomy was reported to show a greater number of down regulated genes.

An independent set of genes that expressed exclusively either in 45,X or 46,XX cells were also retrieved. This includes a total of 31 genes with two antisense transcripts and four lincRNAs. Database for Annotation, Visualization and Integrated Discovery (DAVID) [33] analysis revealed that this set of genes is associated with neurogenesis, cell communication and transcription factor activity. One of these genes is SRY (sex determining region Y)-box 5 (SOX5), which is known to play important roles in chondrocyte differentiation, skeletal development and neuronal development including spermatogenesis [34]–[36]. The detail for this set of genes is summarized in Table S2.

### Functional Analysis of Differentially Expressed Genes

Functional annotations of the differentially expressed genes were carried out using DAVID [33]. We performed the analysis separately for empirical sets defined by a twofold and a fivefold differential expression difference. The gene sets corresponding to a 5 fold differential expression were enriched for the following functional categories; namely bone development (Enrichment score [ES] = 1.2), regulation of cell growth (ES = 1.2) and regulation of transcription (ES  = 0.19) among others. A similar analysis of the gene set showing a 2 fold, differential expression suggested enrichment for functional categories; namely, peptidase activity (ES = 2.9), immune responses (ES = 0.86), apoptosis (ES = 0.51) and transcription repressor activity (ES = 0.37). Several differentially expressed genes (>5 fold difference) map to biological processes responsible for response to steroid hormone stimulus (9%), regulation of carbohydrate metabolism (5%), ossification and bone development (7%) and female pregnancy (4%). Further, many of the genes with a >2 fold expression difference mapped to biological processes related to defence response and adhesion. Similarly, genes that are down regulated (>5 fold) in 45,X condition (compared to 46,XX) were seen to be associated with biological process related to bone development (ES = 1.7), regulation of cell size (ES = 1.38), transcription regulation (ES = 0.11) and also found to be involved in bone development and ossification (12%), glucose import and metabolism (8%), response to hormone stimulus (32%) among others. Detailed summaries of these datasets are available as Table S3 and S4.

### Differentially Expressed Long Noncoding RNAs

Our analysis also revealed differential expression for five lncRNA transcripts in the 45,X and 46,XX fibroblast cells and most of them were down regulated in the 45,X fibroblast cells. This includes one of the well annotated and well-studied lncRNA, XIST that is known to be associated with the X chromosome inactivation. As expected, XIST was found to be down-regulated and had almost no expression in the 45,X cell line as compared to 46,XX fibroblasts. The expression levels and the annotations of the lncRNA transcripts are summarised in Table 2.

**Table 2:** Differentially expressed long non-coding RNAs in 45,X and 46,XX fibroblast cells at P value<0.001 and FDR (q) value<0.05.

Symbol	Chromosomallocation	46,XXFPKM	45,XFPKM	P value	q value
RP11-215N21.1	chr10∶109517590–109871360	0.783563	0	0.00015	0.022832
AP000688.8	chr21∶37377635–37379899	1.85412	0	0.0002	0.027783
SNHG5	chr6∶86370709–86388451	308.543	66.8161	0.0001	0.016259
XIST	chrX:73040485–73072588	20.9437	0	0.00005	0.009291
(MIR663A)RP3-410C9.1	chr20∶26167555–26232162	716.451	0	0.00005	0.009291

### Validation of the Differentially Expressed Genes Using Quantitative Real Time-PCR (qRT-PCR)

**Figure 2:**
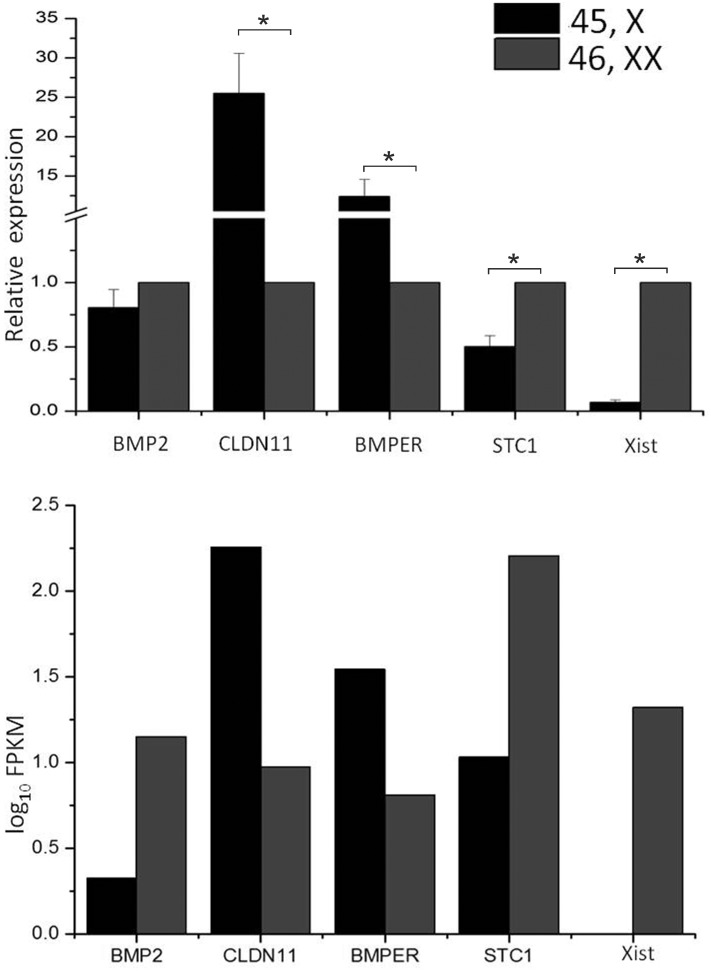
Validation of RNA-seq results by qRT-PCR. In total, five genes including XIST (lincRNA) were selected for real time data validation. Relative expression and FPKM values (log10) are shown for 45,X and 46,XX conditions. qRT-PCR results are found to correlate with RNA seq data, hence further confirming the expression dataset.

qRT-PCR was used to validate the observations obtained from RNA sequencing. We selected five genes for the qRT-PCR analysis. These include genes important in the bone metabolism and gonad development. Except bone morphogenetic protein 2 (BMP2), all the other genes showed significant (P<0.05) change in mRNA expression levels between 45,X and 46,XX cells. Real time data and RNA sequencing data of the selected genes have been summarized in Figure 2.

## Discussion and Conclusions

We have investigated the transcriptome profiles of 45,X and 46,XX human untransformed fibroblast cells using high throughput RNA sequencing technology. RNA sequencing has been extensively used by a number of groups recently, as it provides a genome-scale view of the transcriptome, not limited by the number of designed probes as in others targeted approaches like cDNA microarrays [37]. Previously, Wen Li and colleagues [38] using microarray, have studied global gene expression pattern in a human 45,X fibroblast cells and iPSC. Relatively fewer genes were found to differ amongst the 45,X and euploid iPSCs. A similar study by Zhang *et. al* [24] using RNA sequencing had reported significant number of genes that are differentially expressed amongst these iPSCs. A comparison of our RNA sequencing results with that of the previously published dataset by Zhang *et al.* [24], has identified a significant number (39) of genes common and differentially expressed in both sets. This suggests that common pathways and molecular mechanisms are operating in association with the aneuploidies, notwithstanding the background cell types in which the experiments have been performed (Table S5). Though some of the genes shown to differentially express in the present analysis, is shared with previous studies, one of the caveats of our analysis is that it is confined to differentiated cells, rather than *in-vivo* models. Thus our analysis precludes the potential explanation of specific genes responsible for lethality in 45,X fetuses.

Turner individuals (X monosomy) exhibit an array of clinical phenotypes including osteoporosis, short stature, Diabetes mellitus Type II and gonadal failure. The most accepted hypothesis for Turner phenotypes is the haploinsufficiency of X linked genes like ribosomal protein S4, X-linked (RPS4X) [39], zinc finger protein, X-linked (ZFX) [40] and short stature homeobox (SHOX) [15]. Previous studies on human embryonic stem cells [41] and iPSCs [24] show that X monosomy has a global effect on gene expression, which may be a possible cause for early lethality in most 45,X individuals. We have identified four pseudo-autosomal genes that are expressed at significantly higher levels in 46,XX cells (table S1). Haploinsufficiency of ZFX/Y, RPS4X/Y has been thought to be associated with the Turner syndrome. DEAD (Asp-Glu-Ala-Asp) box helicase 3(DDX3)X/Y gene family is believed to be involved in embryogenesis, spermatogenesis, cellular growth and division while PRKX/Y encodes a protein kinase which may be related to macrophage and granulocyte maturation. Interestingly, all four of these genes have almost no expression in the 45,X karyotype.

Our analysis identified a subset of differentially expressed genes that are associated directly or indirectly with bone metabolism. This potentially suggests a biologically plausible explanation for the high incidence of osteoporosis, which has been one of the characteristic clinical observations in Turner syndrome. One of the genes associated with bone mineralisation, bone morphogenetic protein 2 (BMP2), was found to be down regulated (>6 fold) in 45,X cells. Previous studies on mice [42] and humans [43]–[45] have shown that BMPs are important in bone growth and development. Another gene, BMP binding endothelial regulator (BMPER) was found to have a higher expression in (>5 fold) 45,X condition. This gene encodes a secreted protein that interacts with, and inhibits BMPs function. Genes such as insulin-like growth factor 2 (IGF2), placental growth factor (PGF), prostaglandin-endoperoxide synthase 1 (PTGS1), which are known to have major role in bone repair, formation and development [46]–[52], were found to be down regulated in 45,X cells. In the present analysis, genes such as secreted frizzled-related protein 1 (SFRP1) that is known to be associated with Wnt signaling was also found to be down regulated (>4 fold) in 45,X cells. Altered SFRP1 expression has been shown to affect osteoblast proliferation and differentiation [53], [54]. We also noticed that microfibrillar-associated protein 4 (MFAP4) was up regulated in 45,X cells (>5 fold) and has been associated with Smith- Magenis syndrome [55], a multisystem disorder characterized by phenotypes similar to Turner syndrome such as mental retardation, short stature, obesity, and heart defects. In addition it is likely that, in Turner patients, altered level of sex hormones may influence the bone mineralization and osteoporotic feature.

Functional enrichment analysis also revealed a subset of genes to be closely related to regulation of carbohydrate metabolic process. It is a well established fact that Turner individuals are more susceptible to type II Diabetes mellitus than normal females [28]. Both insulin-like growth factor 2 (IGF-2) [56], [57] and ectonucleotide pyrophosphatase/phosphodiesterase 1 (ENPP1) [58], [59] that have crucial roles in obesity and type II Diabetes mellitus, were found to be down regulated (>22 fold) in 45,X cells, while genes including Tribbles Pseudokinase 3 (TRIB3) involved in insulin signaling [60] showed more (>4 fold) expression in 45,X cells. Turner patients are highly prone to autoimmune linked disorders like coeliac disease, Crohn’s disease, ulcerative colitis, hypothyroidism [61], [62]. It has been suggested that the auto-antibodies may be associated with increased risk of Turner individuals to diabetes mellitus II and hypothyroidism [61]. It is interesting to note that in our data around 14% of differentially expressed genes (>2 fold change) are associated with immune responses, apoptosis and cell death (table S4), suggesting a possible alteration of immune pathways in 45,X karyotype.

Infertility is one of the clinical features of Turner syndrome [63]. Our analysis does not show the differential expression of some of the X linked genes escaping inactivation including SHOX which is known to be involved in gonadal functioning [63]. It is interesting to note that SHOX was also not reported to be differentially expressed in the 45,X iPSCs [24]. In our analysis, autosomal genes like solute carrier family 2 (facilitated glucose transporter), member 14 (SLC2A14), claudin 11 (CLDN11) that have previously been shown to be involved in spermatogenesis were found to be up regulated (>18 fold) in the 45,X cells. Another gene stanniocalcin 1 (STC1), which has a function in paracrine regulation of follicular development [64], [65] was also observed to have a low expression (>14 fold) in 45,X cells, while RASD family, member 2 (RASD2), gene which exhibits low expression in 45,X (>5 fold) condition, has been reported to interfere with the functional activity of thyroid stimulating hormone receptor (TSHR), follicle stimulating hormone receptor (FSHR) and luteinizing hormone/choriogonadotropin receptor (LHCGR) [66].

Apart from protein-coding genes, the genome-scale transcriptome analysis allowed us to look at noncoding RNAs, including the expression landscape of lncRNA expression and their expression landscape associated with the X monosomy. This includes one of the well annotated and studied lncRNA, XIST. XIST is known to be associated with X chromosomal inactivation and is transcribed exclusively from the inactive X chromosome. As expected, in our dataset XIST was expressed in 46,XX fibroblasts while no expression was seen in 45,X cells.

Apart from XIST, we observed a number of differentially expressed lncRNAs. The large intergenic non-coding RNA (lincRNA) RP3-410C9.1 is highly expressed in the 46,XX cells when compared to the 45,X cells. Gene for this lincRNA also encodes miRNA 663a, which is shown to act as a tumour suppressor in gastric [67] and lung [68] cancers. Another lincRNA, SNHG5 is found to be down regulated >4 fold in 45,X cells. Reduced expression of SNHG5 has been shown in breast and prostate cancer [69]–[72]. Few reports indicate that [73], [74] individuals with Turner syndrome have a higher risk of getting different types of cancers. LincRNA AP000688.8, which does not express in the 45,X cells, has been proposed to be related to phenotypes that characterize Down syndrome [75]. Considering the fact that lincRNAs are involved in chromatin modification, gene regulation in *cis* or *trans*, the differential expression of a few lincRNAs in X chromosome monosomy needs to be extensively studied with follow up experimental and in-depth functional assays. This could potentially open up new avenues for understanding biological regulation and potential therapeutic targets for the disease.

We have analysed the genome wide changes in transcriptome of the X monosomic (45,X) fibroblast cells in comparison to normal (46,XX) cells using high throughput RNA sequencing technology. We have used untransformed fibroblast cells to ensure true representation of individual tissue. In our analysis of the two karyotypes, we achieved 97% of sequencing coverage that yielded experimental status of about 58,000 genes. Out of these, 116 genes showed significant difference in expression within the two experimental karyotypes. Our observations manifest that X monosomy affects the transcriptomic profile of the cells and differential regulation of genes in Turner syndrome could be the key features to establish genotype-phenotype relation. Many of the characteristic phenotypes observed in the X monosomy condition could also be putatively correlated to the functions of differentially expressed gene sets. In this study, altered gene expression related to the haploinsufficiency and dosage effect suggests the involvement of critical molecular genetic pathways governing bone formation, glucose metabolism, and gonadal functioning. Our analysis also leads us to find differential expression of lincRNAs that are involved in the potential regulation of tumour suppression. Although the current study is focused on fibroblast cells, in future a tissue specific gene expression profiling will help to establish additional genotype phenotype relation in the 45,X karyotype. It would be of great interest to delineate the molecular genetic mechanisms involved in these processes as it could offer possible ways for better understanding the biomolecular regulation in Turner syndrome individuals [30], [76].

## Materials and Methods

### Cell Maintenance

Human fibroblast cell lines of white population with karyotype 45,X (Cat. no. NA 00857) and 46,XX (Cat. no. ND 29194) were obtained from Coriell Cell Repositories, USA. All fibroblasts were maintained in Dulbecco’s Modified Eagle’s Medium (DMEM, Invitrogen, USA) +10% fetal bovine serum (FBS, GIBCO), and Penicillin (100 U/ml)- Streptomycin (100 ug/ml) (Invitrogen, USA). Cells were grown at 37°C with 5% CO_2_.

### RNA Sequencing and Data Generation

RNA was isolated using Trizol method [77]. The mRNA sample preparation was done by using Truseq mRNA sample preparation kit (Illumina, USA). The isolated RNA was captured using poly-dT magnetic beads and fragmentation was performed under elevated temperature. These RNA fragments were used for cDNA synthesis by random hexamers and Superscript II reverse transcriptase (Invitrogen, USA). Second strand cDNA was prepared by Polymerase I and the overhangs were repaired to blunt ends and single A base overhang was added to the blunt ends to enable adapter ligation with the T overhangs. The product fragments were purified and enriched using adaptor specific PCR. The libraries was amplified on c-Bot (Illumina, USA) to produce clusters and were sequenced by 76×2 paired end on Genome Analyzer IIx (Illumina, USA) as per manufacturer’s supplied protocols. The datasets are available at SRA with IDs SRX503398 and SRX503399.

### Data Analysis

The paired end files generated were trimmed using SolexaQA [78] with Phred quality score of 20. Adapter trimming and length sorting was performed using trimmomatic PE software package [79] and further aligned to the genome (hg19 build of the Human Reference Genome) using Tophat [32]. The alignments were processed and the expression levels were quantitated using Cufflinks [32]. The GENCODE annotation [80] of the transcripts was used as the reference. In order to identify differentially expressed genes, RNA sequencing data was analyzed using Cuffdiff, with (FDR) q value set at <0.05. All the genes which had a fold difference >2 and significant change (FDR value q<0.05) were further examined.

### Functional Analysis of Differentially Expressed Genes

The Database for Annotation, Visualization, and Integrated Discovery (DAVID) was used to identify significantly enriched Gene Ontology categories and for functional annotations of differentially expressed genes [33].

### Quantitative Real Time PCR (qRT-PCR) Assay

Some of the differentially expressed genes were selected for qRT-PCR validation. RNA was isolated from 45,X and 46,XX cell lines using Trizol method. According to manufacturer’s instruction, cDNA was prepared from 1 µg of total input RNA using Superscript II (Invitrogen, USA). qRT-PCR was carried out using SYBR Green master mix (Roche, USA) for detection in Light cycler LC 480 (Roche, USA). We analysed each sample in triplicate, using keratin 5 (KRT5) as an internal control. Statistical analysis was performed with independent samples *t*-test using GraphPad Software. The FPKM values and real time data comparison is summarized in Figure 2. The sequences of the primers used are given in Table S6.

## Supporting Information

Table S1 **List of differentially expressed genes between 45,X and 46,XX human fibroblast cells at q<0.05.**
(XLSX)

Table S2 **List of genes expressed either in 45,X or 46,XX fibroblast cells.**
(XLSX)

Table S3 **DAVID analysis for the genes with >5 fold increased expression in either 45,X or 46,XX cells.**
(XLSX)

Table S4 **DAVID analysis for the genes with >2 fold increased expression in either 45,X or 46,XX cells.**
(XLSX)

Table S5 **Comparison between RNA sequencing data for 45,X iPSC (Ruosi Zhang et. al, 2013) and 45,X fibroblast cells (Current study).**
(XLSX)

Table S6 **List showing primers used for real time validation.**
(XLSX)
